# Enlarging the scenario of site directed ^19^F labeling for NMR spectroscopy of biomolecules

**DOI:** 10.1038/s41598-023-49247-2

**Published:** 2023-12-12

**Authors:** Valentina Vitali, Francesco Torricella, Lara Massai, Luigi Messori, Lucia Banci

**Affiliations:** 1 Magnetic Resonance Center (CERM), University of Florence, Via Luigi Sacconi 6, 50019 Sesto Fiorentino, Italy; 2 Department of Chemistry “Ugo Schiff”, University of Florence, Via Della Lastruccia 3, 50019 Sesto Fiorentino, Italy; 3 Consorzio Interuniversitario Risonanze Magnetiche di Metalloproteine (CIRMMP), Florence, Italy

**Keywords:** Chemical modification, Proteins, Solution-state NMR

## Abstract

The possibility of using selectively incorporated ^19^F nuclei for NMR spectroscopic studies has retrieved increasing interest in recent years. The high gyromagnetic ratio of ^19^F and its absence in native biomolecular systems make this nucleus an interesting alternative to standard ^1^H NMR spectroscopy. Here we show how we can attach a label, carrying a ^19^F atom, to protein tyrosines, through the use of a specific three component Mannich-type reaction. To validate the efficacy and the specificity of the approach, we tested it on two selected systems with the aid of ESI MS measurements.

## Introduction

NMR spectroscopy provides atomic-level information on magnetic active nuclei. In recent years several works showed that the use of the magnetically active fluorine isotope ^19^F represents an important alternative to the standard proton ^1^H biomolecular NMR spectroscopy^1,2^. The 100% natural abundance, the ½ nuclear magnetic spin, and the high gyromagnetic ratio of ^19^F represent the main features that characterize this nucleus^3^. Furthermore, the large chemical shift range of the fluorine nucleus (30 times larger than the ^1^H nucleus) makes it a very sensitive tool for monitoring changes in its surroundings^4^. This nucleus can be easily incorporated in specific labelling sites resulting in a sensitive and powerful, yet not perturbing, probe for large and complex biological systems. Consequently, this allows the characterization in solution of biomolecules of larger molecular size than those studied through ^1^H NMR. In recent times several applications of ^19^F NMR for biomolecular studies such as protein–ligand interaction^5,6^, protein relaxation^7^, aggregation^8,9^, structural and conformational changes^2^, protein folding and unfolding^10,11^ have been reported. Moreover, ^19^F NMR spectroscopy is receiving increased interest even within the in-cell NMR approach, as its less crowded spectra allow to retrieve dynamical and structural information in a reliable way^8,12,13^. On this respect, Pielack et al. recently used ^19^F NMR to describe how the intracellular environment can influence the protein dimerization equilibrium^14^. The possibility of using different ^19^F-labelled amino acids or tags gives the chance to extend the detection of NMR signals even to crowded environments^4^, as well as to exploit ^19^F for other spectroscopic techniques, such as MAS NMR^15,16^ or ENDOR^17,18^. Mainly, two kinds of approaches are used for ^19^F nuclei incorporation in biomolecular systems: the direct expression of ^19^F labelled proteins^19–21^, or the chemical incorporation of specific molecules/labels containing fluorine nuclei^22,23^. Several works showed how the incorporation of specific fluorinated amino acids (e.g.: F-Phenylalanine, F-Tryptophan, F-Tyrosine) or unnatural amino acids^24^ can be relatively easy achieved in *E.coli* cells using modified minimal media supplemented with fluorinated amino acids analogues^19^. Recently, Gotting et. al^25^ presented a novel strategy to synthesize ^13^C/^19^F/^2^H indoles to be used as tryptophan precursors in protein expression. On the other hand, site directed labelling (SDL) represents an attractive and potentially low-cost alternative to the direct labelling during expression, especially because the biosynthetic labelling approach, although well established, presents challenges to be overcome. In addition, the yield of the expressed protein could be significantly reduced due to the intrinsic toxicity of the fluorinated precursors^26^. Post-expression labelling can be achieved on different kinds of residues such as cysteines, lysines or tyrosines, both native or introduced with mutagenesis. Cysteine labelling is one of the most exploited approaches for the post-expression protein labelling and the use of maleimide-based tags opened up a wide variety of experimental approaches for in-cell NMR experiments^27^. A few fluorinated molecules are known to be suitable for site selective cysteine modification. Usually, these molecules are characterized by one or more trifluoromethyl groups covalently attached to a group prone to nucleophilic substitution. 3-bromo-1,1,1-trifluoroacetone (BTFA)^28–30^ and analogues, 2,2,2-trifluoroethanethiol (TFET)^22,23,31,32^ or 4-(perfluoro-tert-butyl)phenyliodoacetamide (PFP)^33^ are among the most used molecules. Other recent and interesting developed fluorine tags were exploited to observe globular proteins directly inside human cells^34^ and to increase the ^19^F chemical shift dispersion^35^. However, cysteine modification can have some drawbacks, including the fact that in a number of proteins cysteines are in the active site and/or coordinate metal cofactors.

A valuable alternative for protein labelling is tyrosine. One of the features that makes tyrosine an interesting labelling site is its average low natural abundance (just above 3%)^36^, making it one of the rarest amino acids in protein sequences. Moreover, being tyrosine a partially hydrophobic residue, its location can range from being deeply buried inside the protein hydrophobic core to being surface exposed. Since the buried ones are far higher in number than the exposed ones, selecting the right reaction conditions, it could be possible to covalently label the relatively rare surface exposed tyrosines in a site-selective way^37^. Several approaches have been proposed, and recently reviewed, for the modification of tyrosine residues^37^. The most relevant ones involve the use of diazonium coupling reactions^38,39^, of diazodicarboxyamides^40,41^, of sulfur/fluoride exchange (SuFEx) chemistry^42^ and of the Mannich-type reaction^43^. This latter reaction targets the phenolic side chain of tyrosine residues on proteins and is one of the oldest methods developed for tyrosine bioconjugation; it has been successfully used both for grafting fluorophores^43^ and synthetic peptides^44^ to chymotrypsinogen.

**Figure 1:**
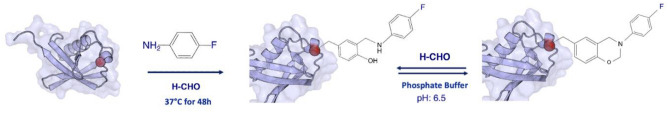
Reaction scheme. General representation of three component Mannich type reaction on tyrosine residue.

The three component Mannich-type reaction (Fig. 1) is characterized by a first step in which an imine condensation between an aldehyde and an electron-rich aromatic amine takes place. Afterwards, the phenol ring of tyrosine is deprotonated and undergoes an electrophilic aromatic substitution with the iminium ion, resulting in the formation of a novel carbon–carbon bond. This reaction was used by Francis et al. to chemically modify proteins using either small peptides or small molecules^43^. Moreover, an interesting application was reported by Belle et al. in which this reaction was used to selectively incorporate a novel spin label for EPR spectroscopy experiments^45^.

In this work we report the protocol for tyrosine protein labelling using parafluoroaniline (p-FA) whose efficacy has been tested, through ESI mass spectrometry and ^19^F NMR measurements, on two proteins of different size.

## Results and discussion

### p-FA tyrosine conjugation

**Figure 2:**
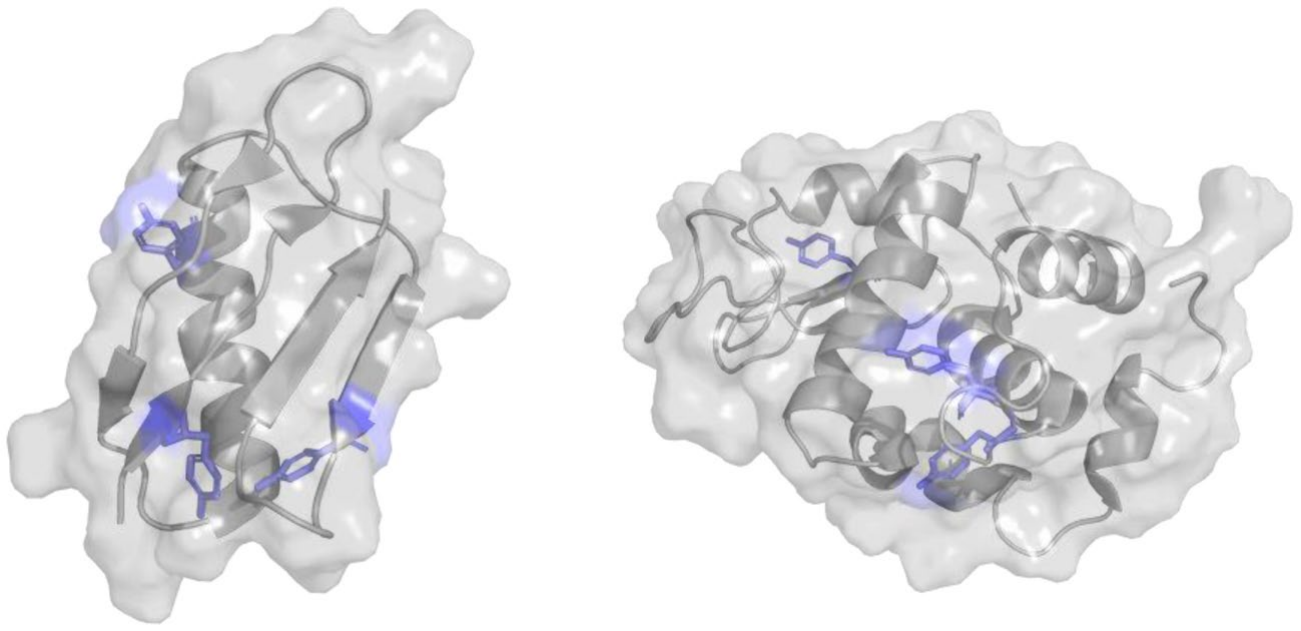
Protein structures. Immunoglobulin binding domain of Streptococcal protein G (GB1) (PDB: 1GB1) and Hen Egg White Lysozyme (HEWL) (PDB: 2VB1). Highlighted in blue their tyrosines.

The immunoglobulin binding domain of Streptococcal protein G (GB1) and Hen Egg White Lysozyme (HEWL) were selected as test proteins (Fig. 2), both proteins having three tyrosine residues, located in different positions of the protein structure.

Both proteins were reacted, exploiting proper reagents ratio, with formaldehyde and the free ^19^F label. The pH at which the reaction is carried out plays a crucial role in the formation of the desired fluorinated tyrosine adducts. Indeed, operating at pH 6.5 is crucial for minimising possible side reactions on unwanted amino acid residues like tryptophans. Moreover, at this pH value the equilibrium that characterizes the reaction, could be driven mostly towards the formation of the open ring Mannich adduct. The reacted samples were then analysed by ^19^F NMR to assess the presence of the fluorinated tag conjugated to the protein tyrosines, and to estimate the overall amount of fluorine nuclei conjugated onto the proteins and the number of tyrosines effectively involved in the conjugation reaction. The attachment of the fluorinated tag was further investigated by ESI–MS spectra of the intact protein before and after the coupling reaction. Mass spectrometry data were used to verify the efficiency of the conjugation reaction and the number of residues to which the tag is attached.

#### GB1

**Figure 3:**
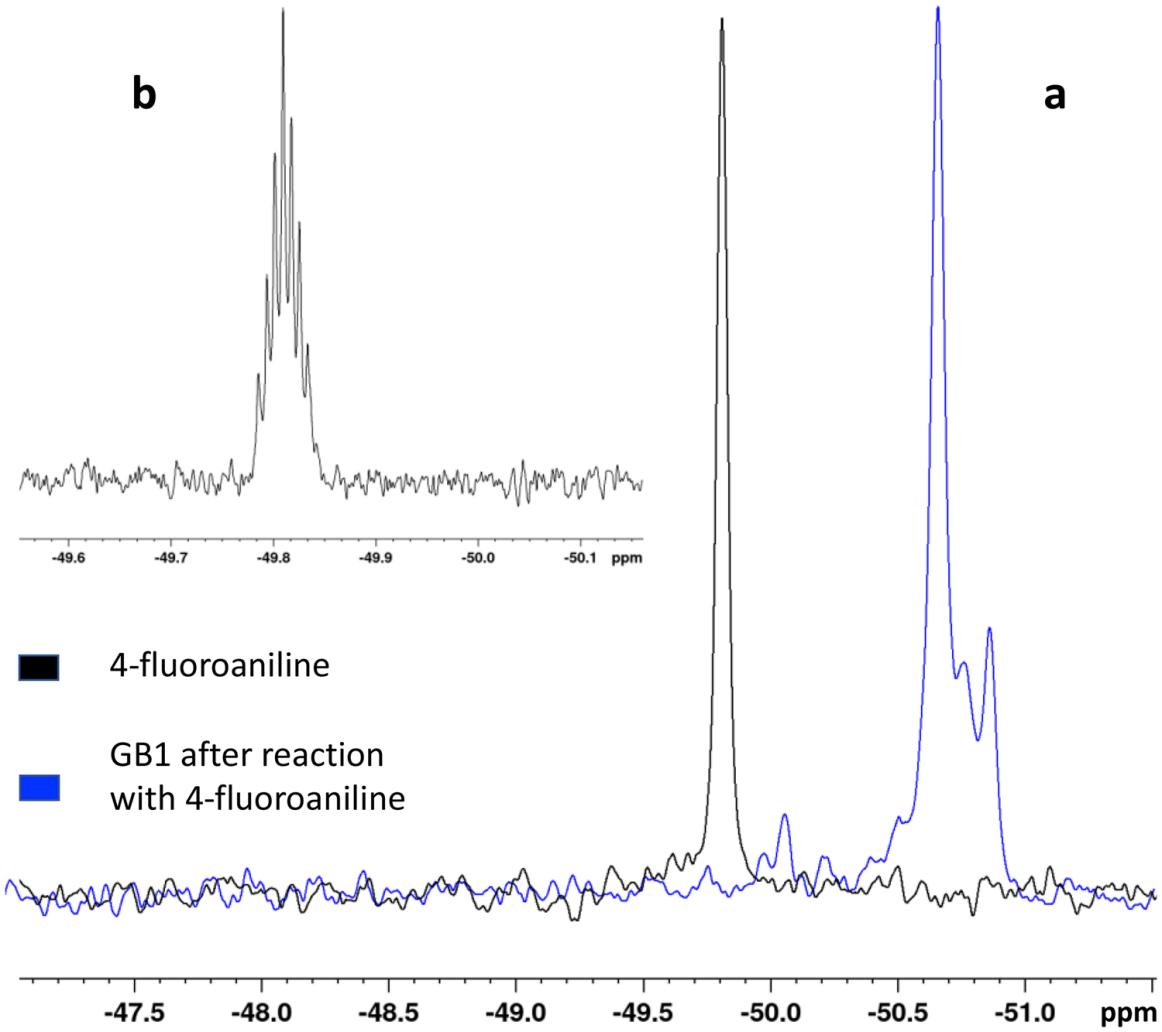
GB1 ^19^F NMR spectrum. (**a**) Comparison between ^19^F NMR spectra of p-FA (black) and GB1 after the conjugation reaction with p-FA (blue). (**b**) 4-fluoroaniline spectrum processed with a line broadening of 1Hz, showing distinct heteronuclear coupling between ^19^F and ^1^H nuclei.

The 1D ^19^F NMR spectrum of GB1 shows the presence of one well defined main peak and two smaller peaks (Fig. 3b), exhibiting different features, both in terms of shape and chemical shift, than the free fluorinated tag signal (Fig. 3a). However, it is impossible to establish the exact number of residues involved in the conjugation reaction by relying just on these NMR spectra.

**Figure 4:**
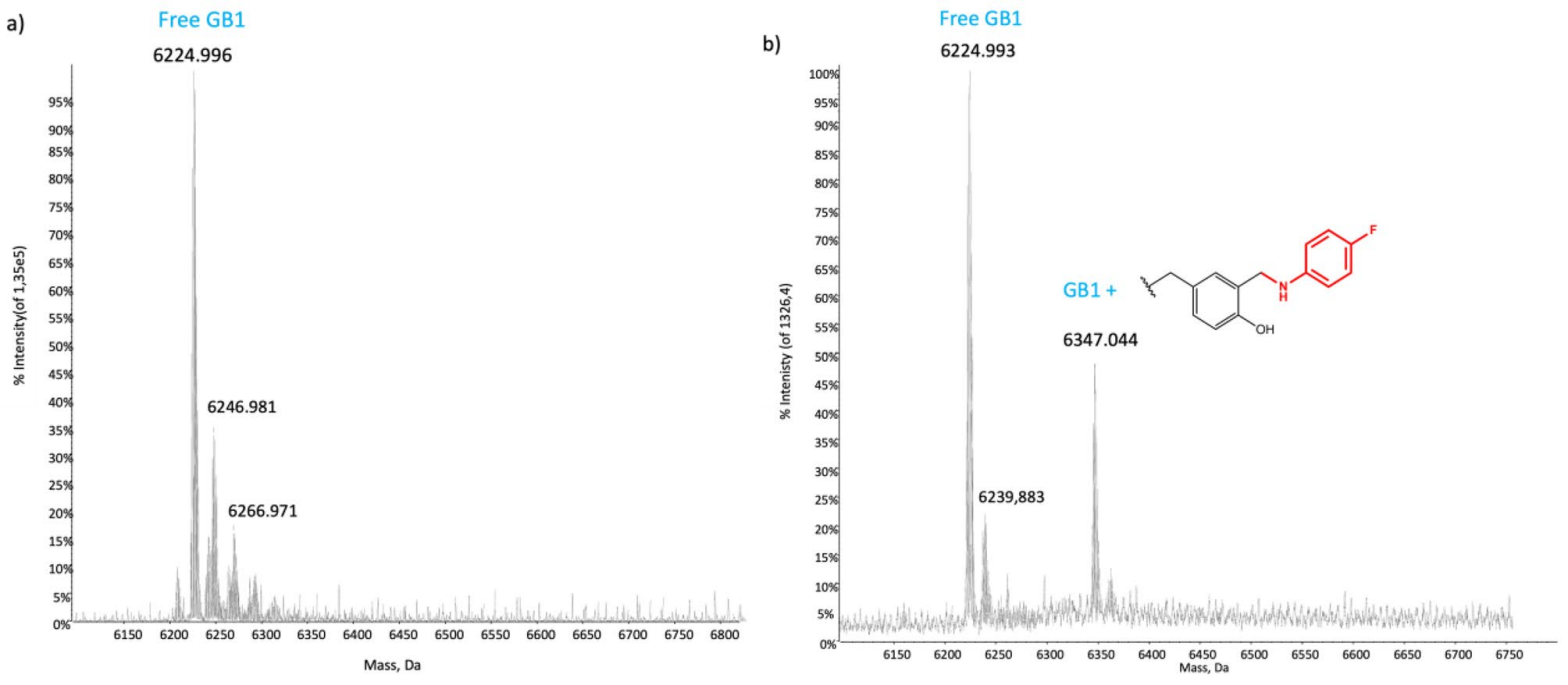
ESI–MS spectra of GB1. Deconvoluted ESI mass spectra of (**a**) GB1, 10^–6^ M in ammonium acetate and (**b**) GB1, 10^–6^ M, after the reaction with fluorinated tag. The peak at 6347 Da represents the GB1 open ring adduct. The bound fragment is red in the drawn structure.

It is feasible that the high intensity signals arise from one labelled tyrosine, but only the ESI–MS spectra clearly indicated (Fig. 4) that a single residue has been successfully labelled. The upfield smaller peaks observed in the ^19^F NMR spectra can be associated with the non-covalent interactions between the protein and a small fraction of p-FA that cannot efficiently be removed during the purification steps of the reaction probably due to π-π stacking interactions between the aromatic ring of the tag and the aromatic rings of other residues.

The mass spectra data (Fig. 4b) indicate that the native unlabelled protein is still the predominant species; yet, a new peak is observed with a mass increase of 123 Dalton (50% intensity of the main peak). This peak originates from GB1 (native unlabelled protein Fig. 4a) with the p-FA tag attached to one residue which, according to the molecular weight increase, is forming the open ring adduct. By assuming an equal ionization efficiency for both species, we can directly assess the ratio of labelled to unlabelled protein which resulted to be 50:100. This high efficiency represents a partial surprise since the Mannich reaction usually employs electron-rich anilines to better attach the carbonyl group of the formaldehyde through a nucleophilic attack. The adduct was obtained through several optimization steps of the reaction conditions, such as the time of the reaction, the temperature, and the ratio of the reagents.

To identify the specific tyrosine modified by the bioconjugation reaction, we performed 1H-13C HSQC NMR spectra on both the native, unlabelled protein and the fluorinated protein. The comparison of the spectra (Supporting Information S3), suggests that the tag is attached to tyrosine 3.

#### HEWL

**Figure 5:**
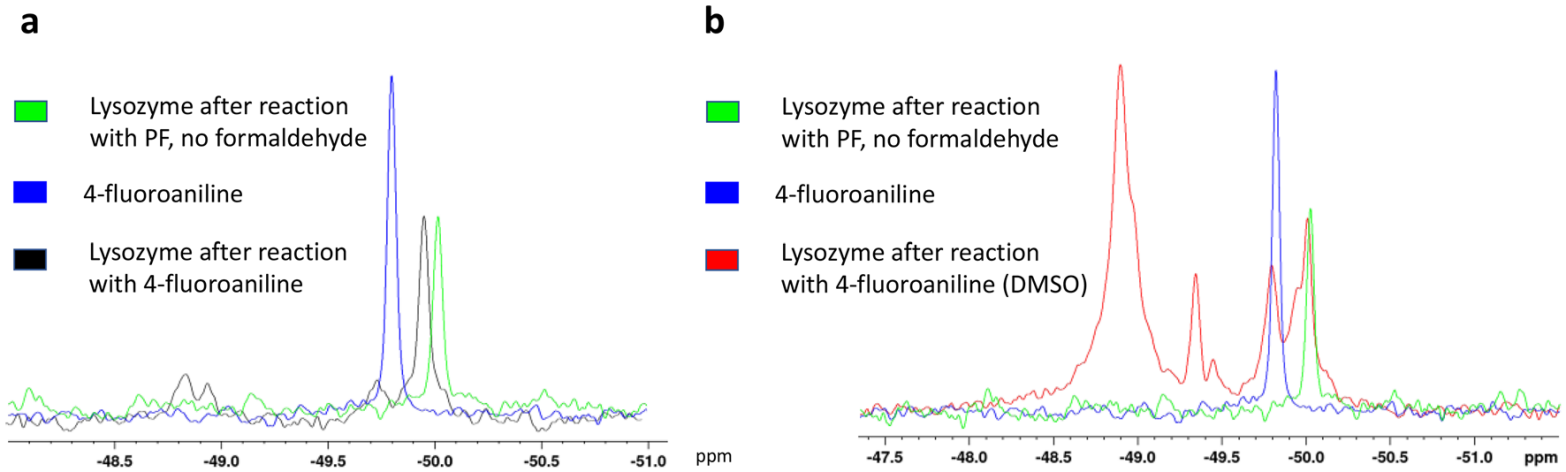
^19^F NMR spectra of lysozyme. (**a**) ^19^F NMR spectra of p-FA (blue), lysozyme after the three component Mannich reaction (black). (**b**) Lysozyme after the three component Mannich reaction without (red) DMSO. The green spectrum represents the non-covalent interactions between the protein and the p-FA.

**Figure 6:**
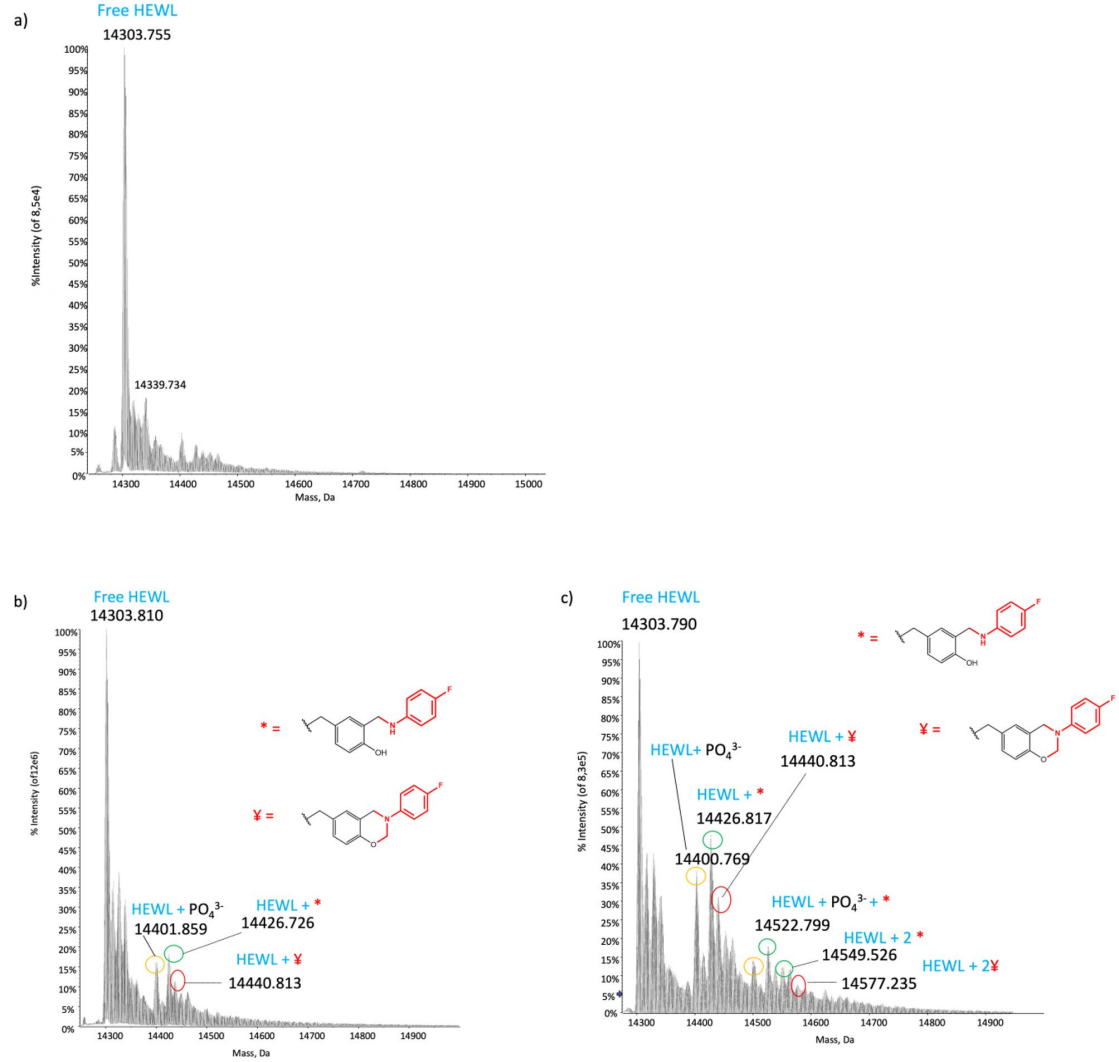
ESI–MS spectra of lysozyme. (**a**) Deconvoluted ESI mass spectrum of free HEWL, (**b**) deconvoluted ESI mass spectrum of HEWL after the conjugation reaction, (**c**) deconvoluted ESI mass spectrum of HEWL after the conjugation reaction with the presence of DMSO. The bound fragments are red in the drawn structures.

A tagged HEWL sample showed a 1D ^19^F NMR spectrum featuring two broad, very weak peaks close to each other at around -49 ppm, and another peak with higher intensity (Fig. 5a) at around -50 ppm. At first glance, this spectrum might suggest that the latter peak originates from an effectively ^19^F-tagged protein tyrosine and that the weaker and broader peaks are due to the low level tagging of the two other tyrosines. However, the intense and sharp ^19^F NMR signal at -50 ppm is detected even for a mixture of the protein and the correct amount of p-FA tag but without addition of formaldehyde (the needed linker between the protein and the fluorinated label) thus indicating that this signal is due to a non-covalent interaction between the fluorinated tag and the protein, while the broad peaks could arise from the tag bound to the protein. Mass spectrometry data (Fig. 6b) confirmed that a single tyrosine among the three of lysozyme (native unlabelled protein Fig. 6a) was modified and that the two distinct broad peaks of the NMR spectrum could originate from a coexistence between the open and closed ring Mannich adduct. The existence of this equilibrium was confirmed through the comparison between simulated and experimental isotopic patterns of the sample under investigation, with peaks at + 123 Dalton for the open ring adduct and at + 137 Dalton for the closed one. Nevertheless, in this case, the reaction efficiency was significantly lower than for the GB1 protein, probably due to the reduced exposure of the tyrosine residues on the protein surface.

To further corroborate this hypothesis, HEWL was treated with 30% DMSO before adding the conjugation reaction reagents. Addition of DMSO induces a partial unfolding of the protein thus increasing the solvent exposition of the residues, including tyrosine, and leading to an increase in the reaction efficiency. The 1D ^1^H NMR spectrum was exploited to confirm the partial unfolding of HEWL after the addition of DMSO (Supporting Information S1). Partially unfolded HEWL was then subjected to the p-fluoroaniline labelling procedure through the Mannich reaction, following the same protocol and time scheme used for the completely folded protein. Its ^19^F NMR spectrum (Fig. 5b) showed significative differences with respect to that of native HEWL. The peaks between − 48.7 and − 49 ppm, already present in the sample without DMSO, are greatly enhanced; a further peak appears at − 49.3 ppm and another broader one with smaller intensity arises between − 49.4 and − 49.5 ppm. These two new peaks hint either at the possibility of labelling a second tyrosine residue or even of a third one, or to have a second tyrosine labelled both with the open and closed ring conformation. The ESI mass data corroborated the existence of the protein labelled just on two different tyrosine residues and for both of them the presence of the open and closed ring adduct, was confirmed. The ESI mass spectrum (Fig. 6c) shows a set of signals similar to those observed after tag binding to the folded protein, but with an overall increase of the labelling efficiency, with a ratio of tagged: untagged of 40:100. Moreover, the existence of a second set of signals, with a lower intensity and a higher molecular weight, confirms the presence of a second tyrosine residue labelled with the fluorinated tag.

Therefore, the use of DMSO allows gaining a larger amount of fluorinated tag attached on the tyrosine residue that is only partially labelled in the absence of DMSO.

These data confirm that only the accessible tyrosine residues can be effectively tagged and that, based on residue exposure, some selectivity in the tagging can be obtained.

## Conclusions

This work presents a different application of an established mild bioconjugation reaction for NMR spectroscopy achieving the labelling of tyrosine residues with a small molecule containing the ^19^F atom. The incorporation of a specific tag containing the ^19^F atom offers the chance to investigate biomolecular systems in less crowded spectra compared to the ^1^H spectroscopy. Moreover, the opportunity to insert ^19^F atoms through a different approach than the direct overexpression of proteins with fluorinated amino acids, provides a different helpful way for situations where the direct overexpression is not applicable. We demonstrated that, using the three component Mannich-type reaction, it is possible to achieve valuable site selectivity among tyrosine residues depending on surface exposure of the tyrosine. The amphiphilicity of the phenolic side chain plays a crucial role in obtaining labelling selectivity, since most of the tyrosines are buried deep in the hydrophobic core and are not available for external modification. Therefore, both the chemical environment and the surface exposure of these residues play an important role in determining whether the residue can be labelled or not. Here, we have demonstrated that, upon adding a given amount of DMSO, HEWL can go from being labelled on one tyrosine to being labelled on two. The denaturating action of dimethyl sulfoxide exposes the residues previously inaccessible and buried inside the protein core, to the solvent and allows the conjugation reaction between the tyrosine and the fluorinated molecule. The use of a commercially available fluorinated tag such as the herein used p-fluoroaniline suggests the possible application of a wide variety of relatively cheap molecules. Moreover, the reaction yield obtained for both GB1 and HEWL with the addition of DMSO should be considered remarkable. In fact, the first step of the three component Mannich-type reaction is an imine condensation between the formaldehyde and the p-fluoroaniline. Since the imine formation starts with a nucleophilic addition of the amine to the carbonyl group, the reaction has a higher efficiency if the amine is electron rich. The fluorine atom is considered an electron withdrawing group (EWG) that decreases the electron density from the nitrogen atom and reduces the efficiency of the nucleophilic attack on the carbonyl group. In conclusion, we have demonstrated how under optimized conditions, a low-cost reaction can be exploited to perform post-expression conjugation of small fluorinated molecules to tyrosine residues. In addition, we established how the protein folding properties play a crucial role in the number of tyrosine residues than can be labelled and even in the efficiency of the reaction towards specific amino acids.

## Materials and methods

### GB1 T53C expression and purification

GB1 was expressed and purified according to already existing protocols^46^. Briefly, a pET-21a vector encoding for the immunoglobulin binding domain of streptococcal protein G (containing the mutation T53C) was used to transform BL21 (DE3) gold cell strain. *E. coli* cells were grown to mid-log phase at 37°C in LB medium, and then induced with 0.6 mM of isopropyl β-D-1-thiogalactopyranoside (IPTG). After induction the cells were grown for other 5 h at 20° C. The cell pellet was collected by centrifugation at 6000 rpm for 20 min and resuspend in phosphate buffer (100 mM sodium phosphate, 150 mM NaCl, pH 6.5). The suspension was heated to 80 °C, for 5 min, using a thermal bath, then cooled down on ice for 15 min and finally centrifuged at 40,000 rpm for 40 min. After filtering the supernatant, 5 mM DTT were added to the solution that was loaded onto a 16/600 Superdex 30 Increase (Cytiva) exchanging the buffer with 100 mM sodium phosphate, 150 mM NaCl, 1 mM TCEP, pH 6.5.

### Hen egg white lysozyme

Hen egg white lysozyme was purchased from Sigma Aldrich.

### ^19^F site directed labelling protocol

Both GB1 and HEWL were reacted with formaldehyde and p-fluoroaniline (both purchased from Sigma Aldrich) with a ratio of 1:100:30 in sodium phosphate buffer 100 mM at pH 6.5. In particular, after thawing, 100 µL of GB1 250 µM were buffer exchanged in the final phosphate reaction buffer using a PD10 desalting column. 50 µL of formaldehyde 0.25M and 30 µL (for each tyrosine) of p-fluoroaniline, were added to the protein solution. Regarding lysozyme, 3.5 mg of protein were resuspended in 1 mL of phosphate buffer for a final concentration of 250 µM. The same amount of formaldehyde and p-fluoroaniline used for GB1 were added to the HEWL solution. A second sample of HEWL was first pre-treated with 30% DMSO and then reacted with formaldehyde and p-fluoroaniline. For both proteins the reaction was incubated at 37 °C for 36h in a shaking incubator. Afterwards, the excess of p-fluoroaniline was removed through a 2.5 mL PD10 desalting column. The 3.5 ml sample volume obtained after passing through the desalting columns was concentrated exploiting a 3KDa, for GB1, and a 10KDa, for the HEWL, centricon (Merck) The volume was reduced until the protein reached 300 µM concentration. However, this method was not able to remove completely the unreacted p-FA tag, especially in the HEWL case. For this reason, a further purification of the labelled lysozyme was conducted by exploiting a gel filtration purification step. Briefly, the sample, in sodium phosphate buffer 100 mM at pH 6.5, was loaded into a size exclusion chromatography Superdex 16/60 75 pg column through a 1 mL loop. The labelled protein was collected in 1.5 mL fractions, that were concentrated to 300 µM. The 1D ^19^F NMR spectrum conducted on this sample confirmed the complete removal of the free unreacted p-FA, (Supporting Information S2). After the purification step small aliquots of each sample were immediately taken and frozen for mass spectrometry (ESI–MS) analysis.

### ^19^F NMR spectroscopy

^19^F magnetic resonance spectra (^19^F NMR) were recorded with a Bruker 600 MHz spectrometer with a TXI probe. Chemical shifts are reported in delta (δ) units, part per million (ppm), and were referenced to trifluoroacetic acid (TFA) as internal standard. 10% of deuterated water was added to the NMR tubes of each sample. All spectra were recorded at 298K.

### MASS-spectrometry

To prepare the samples for mass analysis (ESI–MS), the 100 mM phosphate buffer pH 6.5 was exchanged with 20 mM acetate pH 6.8, with the aim of removing all kind of salts that could interfere with the experimental analysis. For the experiments with GB1 the final protein concentration was 10^−6^ M, whilst for the HEWL was 5 × 10^–7^ M; in all samples 0.1% v/v of formic acid was added just before sample infusion in the mass spectrometer. All measurements were carried out by direct infusion mode without the use of Liquid Chromatography (LC).

Instrumental Parameters: the ESI mass study was performed using a TripleTOF 5600 + high-resolution mass spectrometer (AB Sciex, Framingham, MA, United States), equipped with a DuoSpray® interface operating with an ESI probe. ESI mass spectra were acquired through direct sample infusion at 7 μL/min of flow rate. The general ESI source parameters optimized for the proteins analysis were as follows:

(GB1) positive polarity, ion spray voltage floating 5500V, temperature 25 °C, ion source Gas 1 (GS1) 35 L/min; ion source Gas 2 (GS2) 0; curtain gas (CUR) 20 L/min, collision energy (CE) 10 V; declustering potential (DP) 100 V, acquisition range 900–2000 m/z.

(HEWL) positive polarity, ion spray voltage floating 5500 V, temperature 25 °C, ion source Gas 1 (GS1) 40 L/min; ion source Gas 2 (GS2) 0; curtain gas (CUR) 20 L/min, collision energy (CE) 10 V; declustering potential (DP) 100 V, acquisition range 1000–2800 m/z.

For acquisition, Analyst TF software 1.7.1 (Sciex) was used, and deconvoluted spectra were obtained by using the Bio Tool Kit micro-application v.2.2 embedded in PeakViewTM software v.2.2 (Sciex).

### Supplementary Information

Supplementary Information.

## Data Availability

All data will be available from the corresponding author LB upon reasonable request.
