# Supplementary material for: Enlarging the scenario of site directed 19F labeling for NMR spectroscopy of biomolecules
Source: Sci Rep. 2023 Dec 12;13:22017. doi: 10.1038/s41598-023-49247-2 (PMC10716153; doi:10.1038/s41598-023-49247-2)
Supplement: Supplementary file 1 — Supplementary Information. [file 41598_2023_49247_MOESM1_ESM.pdf]

Enlarging the scenario of site directed ^{19}F labeling for NMR spectroscopy of biomolecules

Valentina Vitali[1,2], Francesco Torricella[1], Lara Massai[2], Luigi Messori[2], and Lucia Banci*[1,2,3]

[1]Magnetic Resonance Center (CERM), University of Florence, via Luigi Sacconi 6, Sesto Fiorentino 50019, Italy; [2]Department of Chemistry "Ugo Schiff", University of Florence, via della Lastruccia 3, Sesto Fiorentino 50019, Italy; [3]Consorzio Interuniversitario Risonanze Magnetiche di Metalloproteine (CIRMMP), Florence, Italy

***Corresponding author**
Lucia Banci: banci@cerm.unifi.it

Table of Contents

1. *GB1 protein sequence* 1
2. *^{1}H 1D NMR spectra of Hen Egg White Lysozyme (HEWL)* 1
3. *Purification after labelling reaction* 1
4. *1H-13CHSQC NMR spectra* 2
5. *Reference* 4

1. GB1 protein sequence

Full amino-acid protein sequence for Immunoglobulin Binding Domain of Protein G, with T53C mutation, where the three tyrosine residues highlighted in red.

```
1         10        20        30        40        50
MQYKLILNGKTLKGETTTEAVDAATAEKVFKQYANDNGVDGEWTYDDATKTFCVTE
```

2. ^{1}H 1D NMR spectra of Hen Egg White Lysozyme (HEWL)

Here reported (**Figure S1**) the two 1D ^{1}H NMR spectra of HEWL before and after the treatment with 30% DMSO. As expected [1], the overall chemical shift dispersion in the ^{1}H NMR spectrum of lysozyme in 30% DMSO, is reduced compared to the native state with broadening of several signals. Moreover, in the region between 5 and 6 ppm we observe a disappearance of peaks which is diagnostic for a partial unfolding state of the protein. We recorder another 1D 1H NMR spectrum after the removal of DMSO and as expected the original folding of the protein was completely restored.

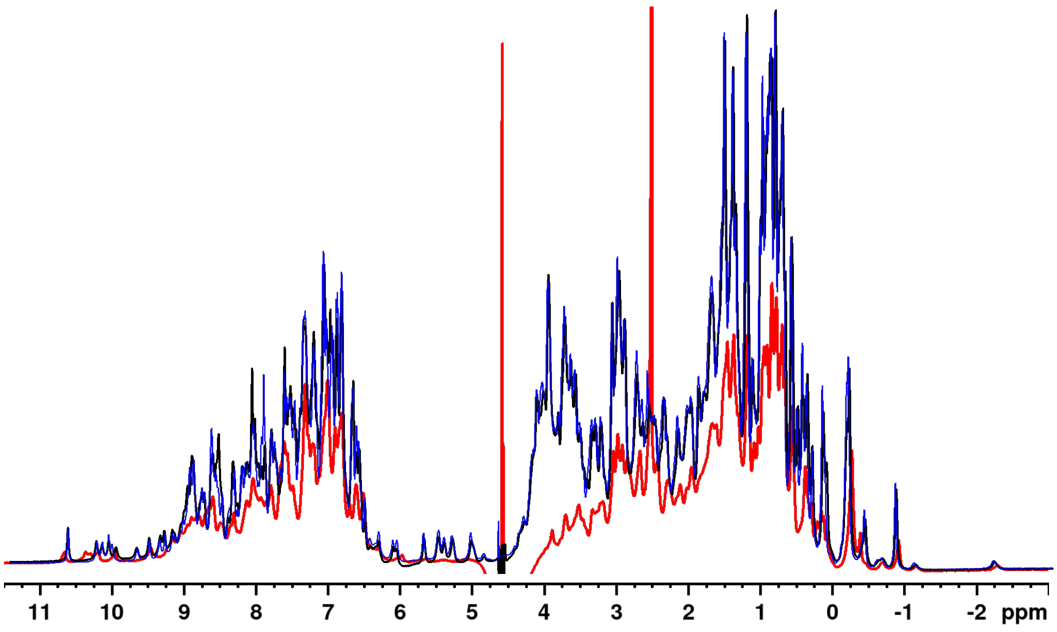

***Figure S1** Lysozyme 1D ^{1}H NMR spectra, before (red) and after (black) 30% DMSO treatment. In blue the spectrum of the HEWL after removing the DMSO.*

All spectra were recorded at 700 MHz, and 25°C with 10% of deuterated water D_2O.

3. Purification after labelling reaction

To enhance the purification step instead of a single PD10 the protein was loaded to a gel filtration column Superdex 16/600 75 pg. The figure below shows the acquired 1D ^{19}F spectrum of the HEWL ^{19}F labelled sample after this additional purification step.

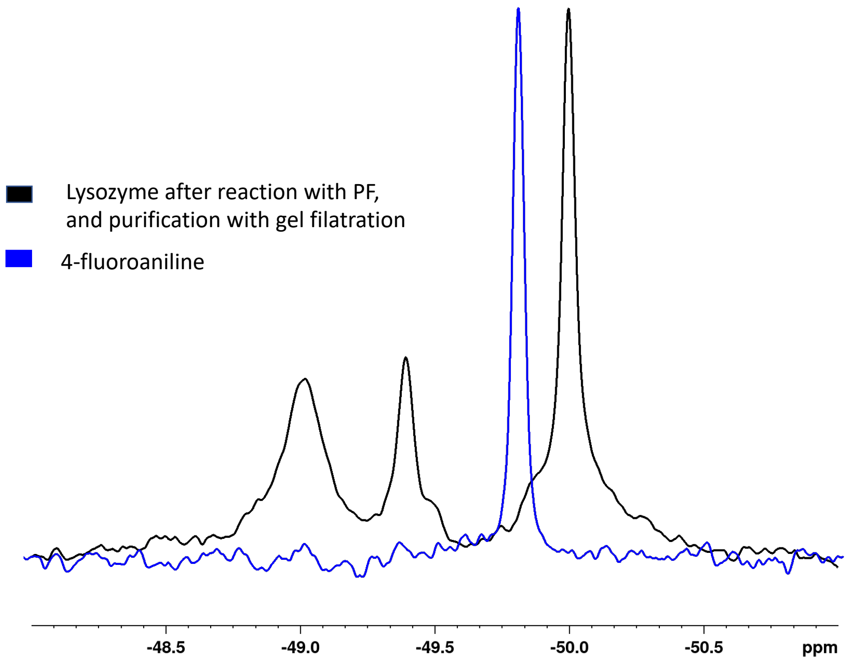

Figure S2 *1D ^{19}F NMR spectrum of HEWL after purification with a size exclusion chromatography column (in black), while in blue the 1D ^{19}F NMR spectrum of the free p-FA label.*

4. 1H-13CHSQC NMR spectra

To determine which of the three tyrosine residues has been labelled in GB1, we acquired 1H-13C HSQC NMR spectra on both the native protein (in red) and the labeled fluorinated protein (in blue) (**Figure S3)**. Our focus was on the aromatic region of the spectrum: as the tag is attached to the epsilon carbon of tyrosine, its signal is expected to be affected. By comparing the normalized intensities of the peaks corresponding to the three tyrosines, we observed a relatively large intensity variation only in the peak associated with the epsilon carbon of tyrosine 3 (**Figure S4).**

The spectra assignment is taken from BMRB code n. 25909

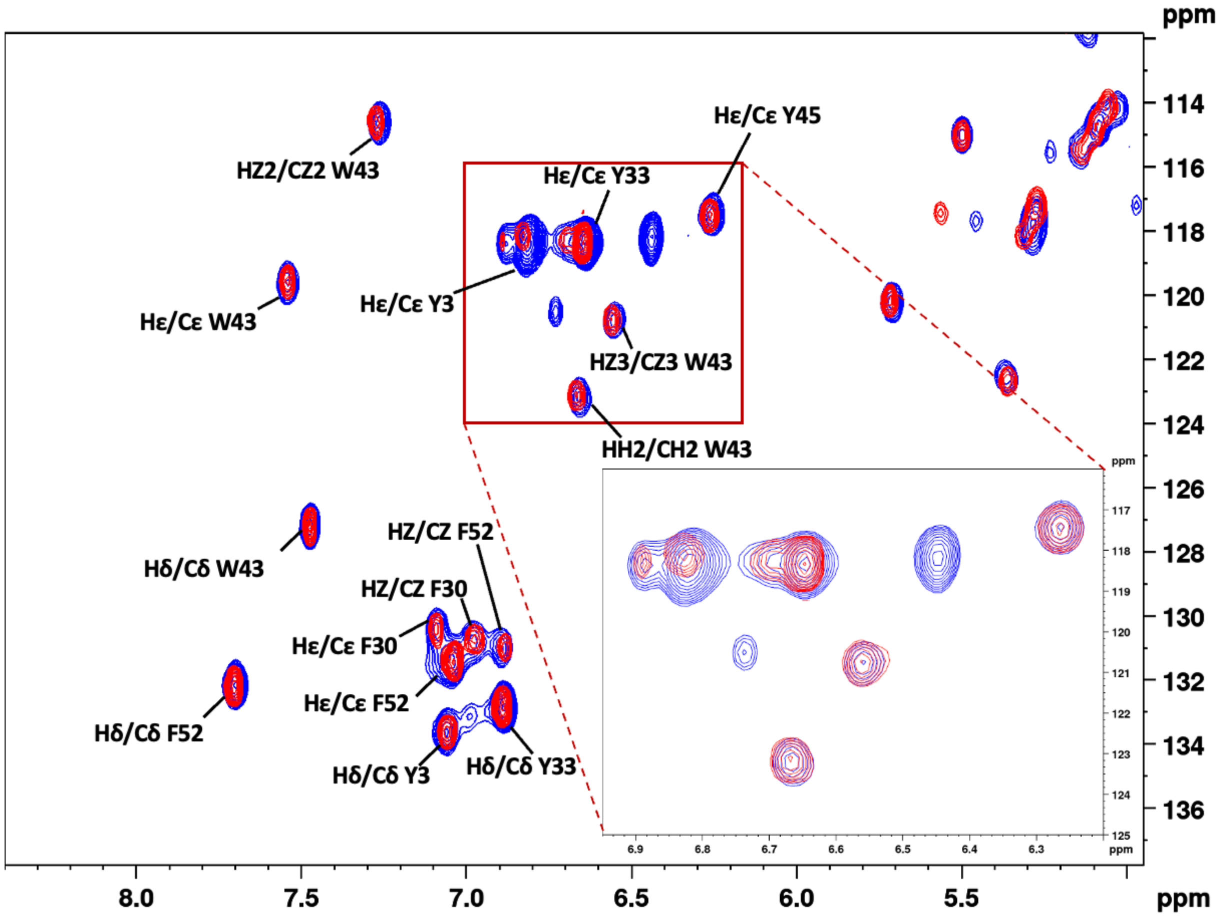

Figure S3 *1H-13C HSQC NMR spectra of the GB1 protein. In red the spectrum of the native protein, in blue the spectrum of the protein after the labelling reaction with the p-FA. The peak belonging to the carbon epsilon of the tyrosine 3. The peaks at 118.20, 6.45 ppm and the one at 120.5 and 6.7 ppm are only present in the blue spectrum and represent the aromatic ring signals of the p-FA tag. All spectra were acquired under the same conditions at 500MHz and 25°C with 10% of deuterated water D_2O for every sample.*

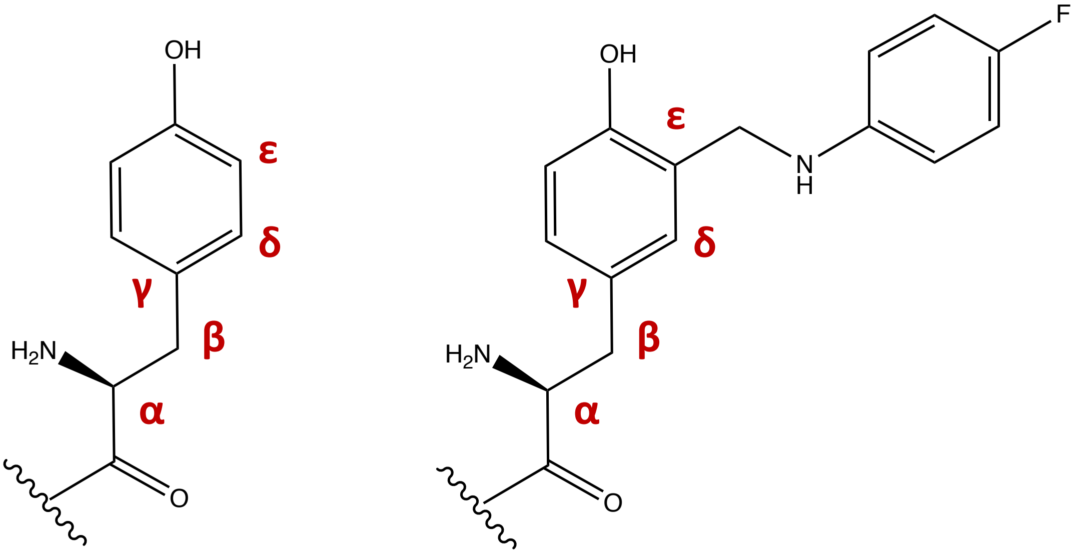

Figure S4 *Schematic representation of the chemical structure of tyrosine residues with and without the fluorinated label. In red the nomenclature used for defining the carbon and hydrogen atoms.*

5. Reference

1 Bhattacharjya, S. & Balaram, P. Effects of organic solvents on protein structures: observation of a structured helical core in hen egg-white lysozyme in aqueous dimethylsulfoxide. *Proteins* **29**, 492-507 (1997). https://doi.org:https://doi.org/10.1002/(SICI)1097-0134(199712)29:4%3C492::AID-PROT9%3E3.0.CO;2-A
